# Effector candidates in the secretome of *Piriformospora indica*, a ubiquitous plant-associated fungus

**DOI:** 10.3389/fpls.2013.00228

**Published:** 2013-07-11

**Authors:** Maryam Rafiqi, Lukas Jelonek, Ndifor F. Akum, Feng Zhang, Karl-Heinz Kogel

**Affiliations:** ^1^Institute of Phytopathology and Applied Zoology, Research Centre for BioSystems, Land use, and Nutrition (IFZ), Justus Liebig UniversityGiessen, Germany; ^2^CeBiTec, Bielefeld UniversityBielefeld, Germany

**Keywords:** fungal effector biology, small secreted proteins, biotrophy, symbiosis, endophyte

## Abstract

One of the emerging systems in plant–microbe interaction is the study of proteins, referred to as effectors, secreted by microbes in order to modulate host cells function and structure and to promote microbial growth on plant tissue. Current knowledge on fungal effectors derives mainly from biotrophic and hemibiotrophic plant fungal pathogens that have a limited host range. Here, we focus on effectors of *Piriformospora indica*, a soil borne endophyte forming intimate associations with roots of a wide range of plant species. Complete genome sequencing provides an opportunity to investigate the role of effectors during the interaction of this mutualistic fungus with plants. We describe *in silico* analyses to predict effectors of *P. indica* and we explore effector features considered here to mine a high priority protein list for functional analysis.

## INTRODUCTION

Plant roots interact constantly with rhizosphere-resident microorganisms. These interactions, which can be either pathogenic or mutualistic, influence plant growth, immunity, and tolerance to abiotic stress (Richardson et al., 2009; Zamioudis and Pieterse, 2012). Beneficial symbioses that supply plants with growth limiting nutrients, such as nitrogen and phosphorus, are of a particular interest to agriculture because they minimize crops requirement for fertilizers. *Piriformospora indica* is a ubiquitous soil borne fungus that associates with roots of a wide range of plant species, including important crops, such as barley and wheat, medicinal plants as well as the model plants *Arabidopsis* and tobacco (Verma et al., 1998; Varma et al., 1999; Rai et al., 2001; Peskan-Berghofer et al., 2004). *P. indica* was initially investigated for its beneficial effects on plant’s growth and resistance to pathogenic infections. Earlier reports have shown that fungal culture filtrates as well as infestation by *P. indica* spores promote shoots growth and increase root branching of plants grown on sterile nutrient-rich media (Barazani et al., 2005; Waller et al., 2005; Deshmukh and Kogel, 2007; Harrach et al., 2007; Serfling et al., 2007), suggesting possible induction of long distance hormonal signals rather than nutrient supply by the fungus. Indeed many microorganisms produce phytohormones or their analogs that induce plants growth and modify root structures (Grunewald et al., 2009). However, recent studies report that while *P. indica* indeed produces auxin during association with *Arabidopsis* and barley roots, fungal auxin production was not found to be required for triggering plant’s growth (Vadassery et al., 2008; Hilbert et al., 2012; Nongbri et al., 2012). More studies are needed to specify the role of hormonal signals mediating the interaction between *P. indica* and plants. While accumulated evidence supports a mutualistic association between plants and *P. indica*, and suggests the use of this fungus as a biocontrol agent, the exact molecular process underlying the antagonistic effect of *P. indica *on pathogenic infections is unknown.

*Piriformospora indica *is a facultative saprophyte that grows on dead plant material and colonizes living root cells, mostly biotrophically, though a switch to a late cell death-associated stage has been described (Deshmukh et al., 2006; Qiang et al., 2012). This late growth stage is symptomless and poorly characterized. Whether this transition in the lifestyle affects mutualistic interactions with plants is as yet unknown. In general, biotrophic fungi have a narrow host range. *P. indica* forms associations with roots of a large range of plant species. Although it is still unclear if these interactions are mutualistic or more parasitic, an intriguing question is what are the cellular and molecular mechanisms developed by this fungus to ensure biotrophic growth and to undermine host defense strategies in different plant species? One scenario is that *P. indica* deploys an effector repertoire targeting conserved cellular processes in many plant species.

**FIGURE 1:**
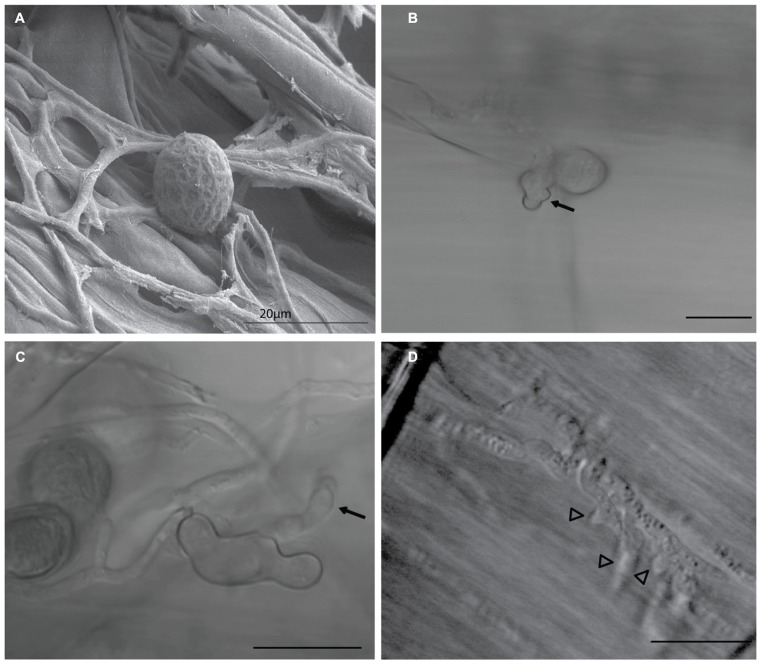
***Piriformospora indica *biotrophic hyphal structures.** During biotrophic growth on barley root cells, *P. indica* spores attach to the root surface, as seen **(A)** germinate and extend intercellular hyphae (arrows) on root tissue within 10 h **(B,C)** Differentiated swollen hyphal structures (arrowheads) are extended into colonized living cells of root tissue **(D)** These structures are morphologically analogous and may share similar functions to haustoria and arbuscules formed by pathogenic and mycorrhizal fungi, respectively, suggesting possible roles in acquisition of nutrients and secretion of effectors into host tissue. Image **(A)** was taken using scanning electron microscope (SEM), Images **(B–D)** were taken using a light microscope. Bars =20 μm.

Key feature of the virulence of many biotrophic and hemibiotrophic fungal pathogens is the ability to deliver virulence proteins called effectors into their host cells. These effector proteins manipulate the host immunity, physiology, and metabolism, in favor of fungal growth and disease development. Some secreted fungal effectors exert their action extracellularly, in the plant apoplastic space. Many others have their molecular targets inside the plant cell, in the cytoplasm, the nucleus or other host subcellular compartments (Rafiqi et al., 2012). During biotrophic growth on barley root cells, *P. indica* intercellular hyphae extend differentiated branched hyphal structures into infected cells of root tissue (**Figure 1**). These structures are morphologically analogous and may share similar functions to the haustoria and arbuscules formed by pathogenic and mycorrhizal fungi, respectively. *P. indica *biotrophic hyphal structures penetrate the cell wall and invaginate the plasma membrane of infected barley root cells, suggesting possible roles in acquisition of nutrients and secretion of effectors in host tissue, similar to haustoria and arbuscules (Voegele and Mendgen, 2003; Catanzariti et al., 2006; O’Connell and Panstruga, 2006; Corradi and Bonfante, 2012). In this review, we use the whole genome sequence of *P. indica *(Zuccaro et al., 2011) to generate a refined list of effector candidates in the secretome of this endophytic fungus.

## IDENTIFYING EFFECTOR CANDIDATES OF *P. indica*

**FIGURE 2:**
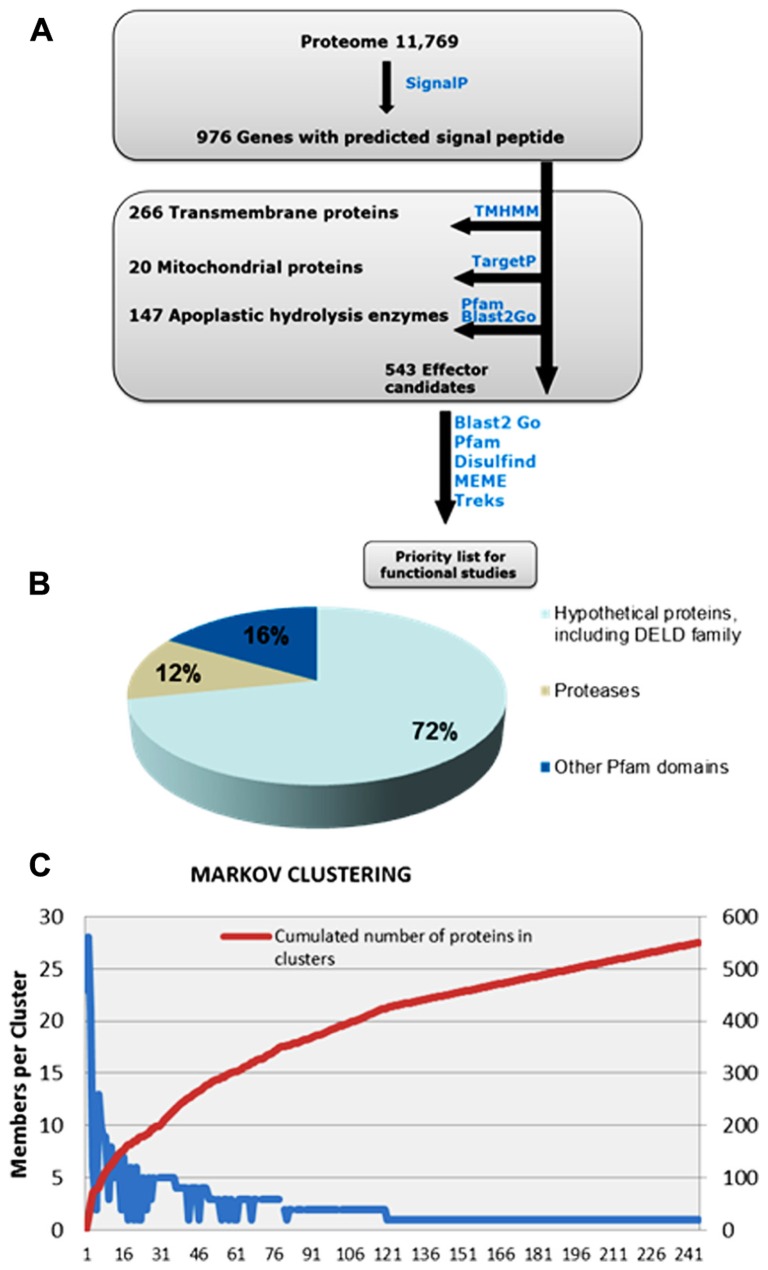
** Overview of the computational pipeline used to mine the list of effector candidates in the secretome of *P. indica*.** **(A)** *P. indica *secretome, consisting of 972 proteins, was predicted using SignalP. Proteins containing transmembrane domains and proteins with mitochondrial signals were removed usingTMHMM and TargetP, respectively. Apoplastic hydrolysis enzymes, such as chitinases and glucanases, were removed based on their function and not on their size, using Pfam and Blast2Go. The remaining total number of 543 proteins are considered effector candidates. Notably, 72% of effector candidates are novel sequences of unknown function **(B)** MCL analysis **(C)** has resulted in a high number of singletons and has shown no evidence for gene clustering.

Recent work on predicting effector candidates from fungal genomes has relied on selecting fungal genes up-regulated during *in planta* growth and coding for predicted small secreted proteins (SSPs) with a size cut-off of 300 amino acids (aa) that do not code for known functions (Martin et al., 2008; Hacquard et al., 2012; Zuccaro et al., 2011). However, more recent research has shown that fungal and oomycete effectors can exceed the size of 300 aa (Rafiqi et al., 2010; van Damme et al., 2012), and that despite being under high selective pressure, some effectors can still carry recognizable Pfam domains, which would help predict their biological function. Examples of these effectors are CRN8 of *Phytophthora infestans* and AvrM of *Melampsora lini*. CRN8 is 600 aa in size and carries a serine/threonine RD kinase domain that has been shown to function in the plant nucleus. AvrM is a 343 aa avirulence protein that is intercepted by the tonoplast-resident flax resistance protein M (Catanzariti et al., 2006; Takemoto et al., 2012; van Damme et al., 2012). Similarly, Ecp6 of *Cladosporium fulvum* and Slp1 of *Magnaporthe oryzae* carry LysM domains, (de Jonge et al., 2010; Mentlak et al., 2012). Thus, for identification of *P. indica* effector protein candidates, we established an *in silico* pipeline that does not take in account protein size and that includes Pfam domain-containing proteins (**Figure 2**).

Using SignalP (Petersen et al., 2011), 976 genes were predicted to code for proteins with signal peptide. Sequence similarity search was run using BlastP. Secreted proteins with predicted apoplastic functions, such as cell wall hydrolysis, were excluded from this set based on their function and not on their size, and proteins with Pfam domains suggesting possible intracellular functions were retained. This resulted in a reduced set of 543 secreted proteins that are considered effector candidates (**Figure 2**). The majority, 389 proteins, are with unknown functions, a feature that characterize many predicted fungal effectors. 154 proteins carry predicted Pfam domains, of which 64 are predicted to have protease activity and 23 carry the carbohydrate-binding protein domain LysM. Effector protein families with LysM domains are expanded in many fungal species and are predicted to contribute to fungal virulence through binding to chitin oligosaccharides, and subsequently preventing their hydrolysis by plant chitinases (de Jonge and Thomma, 2009; Gan et al., 2012; Mentlak et al., 2012) and/or their recognition by membrane-anchored pattern recognition receptors (PRRs) such as *Arabidopsis* chitin elicitor receptor kinase (AtCERK1) that binds chitin directly through its extracellular LysM-containing domain (Liu et al., 2012).

## *Piriformospora indica* EFFECTOR CANDIDATES WITH NO Pfam DOMAIN ARE ENRICHED FOR CYSTEINE RESIDUES AND INTERNAL REPEAT-RICH SEQUENCES BUT SHOW NO EVIDENCE FOR CLUSTERING

132 of the 389 SSPs lacking Pfam domains are enriched for cysteine residues, of which 65 are predicted by Disulfind algorithm (Ceroni et al., 2006) to have three or more disulphide bonds. 14 SSPs showed similarity to predicted proteins in the secretome of *Laccaria bicolor*. Using T-REKS program (Jorda and Kajava, 2009), 110 SSPs lacking Pfam domains were found to contain internal repeat-rich sequences. Search for conserved motifs (RxLR, [L/I]xAR, [R/K]CxxCx12H, [Y/F/W]xC, YxSL[R/K], and G[I/F/Y][A/L/S/T]R) showed no evidence for the presence of conserved motifs identified in SSPs of other fungal and oomycete species. Some of these motifs were present in one or a few sequences. However, because of their low frequency and their short sequences when compared to the more complex SSPs sequences, we consider their presence to occur by random chance. Using the Markov-Cluster-Algorithm (MCL; ) and MCL-Tribe (Enright et al., 2002), 215 SSPs could be clustered into tribes with five or more proteins (**Figure 2**). The remaining 328 sequences were split into 212 smaller clusters, including 138 singletons, and showed no evidence for gene clustering. Among SSPs rich in small repeats, 25 effector candidates carry the conserved C-terminal motif RSIDELD (Zuccaro et al., 2011). The function of this motif is as yet unknown. One new DELD gene (deposited to NCBI GenBank under the accession number KC342232.1) that was missing in the *P. indica* genome database, likely due to the presence of repetitive sequences, was amplified by PCR, indicating that DELD protein family might be more expanded than *ab initio* deduced from the assembled genome. Homologs of DELD proteins are also conserved in the closely related sebacinalean fungus *Piriformospora williamsii* (Rafiqi, unpublished). Proteins of this family have related sequences enriched for alanine and histidine residues and may have expanded from a single ancestral sequence. With the exception of DELD proteins and 14 other SSPs showing similarity to predicted secreted proteins of *L. bicolor*, the majority of *P. indica *SSPs are novel sequences showing no significant homology to known sequences in other organisms, which is in accord with previous studies highlighting the evolutionary diverse nature of fungal effectors (Saunders et al., 2012).

## FAMILIES OF EFFECTOR CANDIDATES WITH PREDICTED INTRACELLULAR FUNCTIONS

Among Pfam-containing effector candidates, 35 indicate intracellular regulatory functions, suggesting that they perform these functions after translocation into plant root cells. Examples of these predicted intracellular effectors are translation activators, RNA-binding proteins, RING fingers and F-box-containing proteins that are involved in protein ubiquitination. In addition, 14 SSPs with no Pfam domains carry predicted nuclear localization signals (NLSs). *In planta* expression of three green fluorescent protein (GFP)-tagged NLS-harboring proteins lacking the signal peptide resulted in nuclear localization of GFP fusion proteins, confirming the functionality of the NLS in plant cells and presenting indirect evidence for the intracellular function of these effector candidates (Rafiqi, Unpublished). Effectors with predicted intracellular functions constitute a high priority list for further analysis of the biological role as well as the translocation mechanism of fungal effectors in plant cells. Preliminary yeast two hybrid screen results indicate interaction of one of *P. indica *effector candidates with CSN5a and CSN5b components of the COP9 signalosome in *Arabidopsis* and tobacco, and with a member of *Arabidopsis* stress-associated protein family (AtSAP) that act as E3 ligase (Boernke and Rafiqi, unpublished). CSN5 is an evolutionary conserved protein complex comprised of eight subunits, named CSN1-8, where CSN5 is the only catalytic subunit described so far. CSN5 is an isopeptidase that interferes with the ubiquitin-proteasome pathway and plays critical developmental roles in plants (Wei et al., 2008). Targeting both CSN5 and AtSAP gives molecular insights into how *P. indica* could manipulate protein ubiquitination in different plant species by targeting conserved molecular processes in plants.

Unlike pathogenic cytoplasmic effectors, which can be revealed through a screen for avirulence functions in resistant plants, mutualistic cytoplasmic effectors are more challenging to identify. In a recent study, Kloppholz et al. (2011) have used yeast secreted protein trap system to identify a cytoplasmic effector, SP7, of the arbuscular mycorrhiza, *Glomus intraradices*. SP7 that target the plant nucleus is thought to promote symbiotic biotrophy through interaction with the plant transcription factor ERF19 that inhibit host defenses during mycorrhization. Similarly, another cytoplasmic effector, MiSSP7, that enters the plant nucleus and alters host gene expression was identified in the genome sequence of the ectomycorrhiza *L. bicolor *(Plett et al., 2011). Cell death suppression is likely to be a redundant function in the effector repertoire of mutualistic fungi.

Besides their biological function, how *P. indica* cytoplasmic effectors enter host cells is an important question to address. Translocation of fungal effectors is a topic of great debate. Evidence has been presented that translocation of some oomycete and fungal effectors, including two mutualistic effectors MiSSP7 of *L. bicolor* and SP7 of *G. intraradices*, can be pathogen-independent (Kale et al., 2010; Rafiqi et al., 2010; Kloppholz et al., 2011; Plett et al., 2011). However, the question of how fungal effector proteins reach the cytoplasm of plant cells is still widely debated.

## PERSPECTIVES

As more and more evidence comes in to support the biological role of fungal effectors in manipulating plant immunity in favor of fungal virulence, selecting biologically significant proteins among hundreds of predicted effector candidates revealed by genome sequencing, and establishing a priority list for functional analysis remain critical. Isolation of *P. indica *biotrophic hyphal structures and construction of complementary DNA (cDNA) library of genes that are differentially expressed in these structures are necessary to identify effectors deployed at different stages of fungal morphogenesis. Available transcriptome sampled from colonized roots masks the expression pattern of *in planta* induced genes due to abundant extracellular and saprophytic mycelia, and to the low ratio of fungal to plant biomass in the early stages of root colonization. An important question is how *P. indica* evades recognition by the plant surveillance system, and whether it switches from restricted mutualistic to proliferative parasitic or pathogenic growth. Investigating the biological activity of effector proteins may provide mechanistic insights into how *P. indica* colonizes plants, at the molecular level.

## Conflict of Interest Statement

The authors declare that the research was conducted in the absence of any commercial or financial relationships that could be construed as a potential conflict of interest.
